# Hilbert–Schmidt speed as an efficient figure of merit for quantum estimation of phase encoded into the initial state of open *n*-qubit systems

**DOI:** 10.1038/s41598-021-86461-2

**Published:** 2021-03-29

**Authors:** Hossein Rangani Jahromi, Rosario Lo Franco

**Affiliations:** 1 Physics Department, Faculty of Sciences, Jahrom University, P.B. 74135111, Jahrom, Iran; 2 Dipartimento di Ingegneria, Università di Palermo, Viale delle Scienze, Edificio 6, 90128 Palermo, Italy

**Keywords:** Quantum information, Quantum mechanics, Quantum metrology, Qubits

## Abstract

Hilbert–Schmidt speed (HSS) is a special type of quantum statistical speed which is easily computable, since it does not require diagonalization of the system state. We find that, when both HSS and quantum Fisher information (QFI) are calculated with respect to the phase parameter encoded into the initial state of an *n*-qubit register, the zeros of the HSS dynamics are actually equal to those of the QFI dynamics. Moreover, the signs of the time-derivatives of both HSS and QFI exactly coincide. These findings, obtained via a thorough investigation of several paradigmatic open quantum systems, show that HSS and QFI exhibit the same qualitative time evolution. Therefore, HSS reveals itself as a powerful figure of merit for enhancing quantum phase estimation in an open quantum system made of *n* qubits. Our results also provide strong evidence for both contractivity of the HSS under memoryless dynamics and its sensitivity to system-environment information backflows to detect the non-Markovianity in high-dimensional systems, as suggested in previous studies.

## Introduction

The process of quantum parameter estimation typically consists in preparing the probe system in an initial state, letting it interact with the target system to encode the information about the unknown parameter, then measuring the probe to extract the information and finally estimate the parameter. It should be noted that in this process, the system also may be affected by different noises. Provided that the physical mechanism governing the system dynamics is known, one may deduce a value of the parameter, with a given precision, by comparison between the input and the output states of the probe^1,2^.

Phase estimation is at the heart of quantum metrology^2–15^ due to the fact that, in many technological areas, the parameter estimation problem can be reduced to determining a phase shift induced in the quantum state of the probe. Measuring an unknown phase has many significant applications in different scenarios, some of them being gravitational wave observation^16–19^, test-mass position measurements^20^, frequency standards^21^, gravimeters^22,23^ and detection of weak signals or defects resulting in the design of very sensitive sensors^24^. In these scenarios an interferometric scheme is typically used to implement the quantum phase estimation, as happens for optical interferometry in gravitational wave detectors^25^, atomic interferometry in Ramsey spectroscopy and physical law testing^13^, optical imaging or laser gyroscopes^2^ to name a few. All of these applications usually aim at optimal estimation of a relative phase gathered by the signal along one arm of the interferometer^26^.

According to the quantum Cramér-Rao theorem, the precision of quantum phase estimation is bounded by the inverse of the quantum Fisher information (QFI)^4,27–29^, which thus represents a central quantity in quantum metrology. In fact, evaluation of the QFI provides the ultimate quantum limits to measurement precision and consequently a general benchmark to assess quantum metrological protocols. The QFI is also a measure of quantum statistical speed that quantifies the sensitivity of an initial state with respect to changes of the parameter which should be estimated. In general, each measure of statistical distance naturally leads to a statistical speed for parametric evolutions of classical probability distributions or quantum states. This statistical speed can be obtained by the change in distance originated from a small variation of this parameter (i.e., the derivative of the distance). The quantum statistical speed is then obtained by maximizing over the classical statistical speed over all quantum measurements^30^.

Inspired by the fact that the QFI is a measure of quantum statistical speed, obtained from the Hellinger distance^31^, here we investigate the application of the Hilbert–Schmidt speed (HSS), another quantifier of quantum statistical speed, in the process of quantum phase estimation. Because calculating the QFI for high-dimensional quantum systems is typically complicated^32^, it is useful to inquire the efficiency of the HSS, which is an easily computable quantity having the advantage of avoiding diagonalization of the evolved density matrix, in the quantum estimation theory. In this paper, we show that the HSS can be indeed exploited as a powerful and convenient figure of merit in quantum metrology for *n*-qubit systems. This result gains particular attention considering the fact that many quantum information protocols are designed by *n*-qubit registers.

## Results

### Preliminaries: quantum Fisher information (QFI)

We start our analysis by recalling the general formulation leading to defining a kind of quantum statistical speed by which the quantum Fisher information (QFI) can be characterized.

First, we consider the (classical) Hellinger distance^31^

1 $$\begin{aligned} {[}d(p,q)]^{2}=\dfrac{1}{2}\sum \limits _{x}^{}|\sqrt{p_{x}}-\sqrt{q_{x}}|^{2}, \end{aligned}$$

where $p = \{p_{x}\}_{x}$ and $q = \{q_{x}\}_{x}$ represent the probability distributions, i.e., probabilities of occurrence of different possible outcomes for two different experiments. Here the random variable *x* is assumed to take only discrete values. Formally, in order to achieve the statistical speed from a given statistical distance, one should quantify the distance between infinitesimally close distributions taken from a one-parameter family $p_{x}(\varphi )$ with parameter $\varphi$. In other words, the statistical speed versus a special parameter is defined as the change in the corresponding distance originated from a small variation of the parameter. Following this prescription and performing a Taylor expansion at $\varphi _{0}$ for small values of $\varphi$, we find that the classical statistical speed associated with the (classical) Hellinger distance is given by

2 $$\begin{aligned} s[p(\varphi _{0})]\equiv \dfrac{{\text{d}}}{{\text{d}}\varphi }d\big (p(\varphi _{0}+\varphi ),p(\varphi _{0})\big )=\sqrt{\dfrac{f(p(\varphi _{0}))}{8}}, \end{aligned}$$

where

3 $$\begin{aligned} f(p(\varphi ))=\sum _{x}^{}p_{x}(\varphi )\bigg (\dfrac{\partial ~ \text {ln}p_{x}(\varphi )}{\partial \varphi }\bigg )^{2}, \end{aligned}$$

denotes the Fisher information^4,28^.

Extending these classical notions to the quantum case and considering a given pair of quantum states $\rho$ and $\sigma$, one may write $p_{x} = \text {Tr}\{E_{x}\rho \}$ and $q_{x} = \text {Tr}\{E_{x}\sigma \}$ representing the measurement probabilities associated with the positive-operator-valued measure (POVM) defined by the set $\{E_{x}\ge 0\}$ which satisfies $\sum _{x} E_{x} = {\mathbb {I}}$. The associated quantum distance can be obtained by maximizing the (classical) Hellinger distance over all possible choices of POVMs^33^, that is

4 $$\begin{aligned} D(\rho ,\sigma )=\max _{\{E_{x}\}}d(p,q)=\sqrt{1-{\mathcal {F}}(\rho ,\sigma )}, \end{aligned}$$

called the Bures distance^28^ in which ${\mathcal {F}}(\rho ,\sigma )\equiv \text {Tr}\sqrt{\sqrt{\rho }\sigma \sqrt{\rho }}$ denotes the state fidelity^34^. Now, the quantum statistical speed^28^ can be defined as

5 $$\begin{aligned} S[\rho (\varphi _{0})]\equiv \dfrac{{\text{d}}}{{\text{d}}\varphi }D \big (\rho (\varphi _{0}+\varphi ),\rho (\varphi _{0})\big ) =\sqrt{\dfrac{F(\rho (\varphi _{0}))}{8}}, \end{aligned}$$

in which the QFI $F(\rho (\varphi ))$ appears and is given by^1,5,6,28^

6 $$\begin{aligned} F(\rho (\varphi ))\equiv F_{\varphi }=\sum _{i,j}\frac{2}{\lambda _{i}+\lambda _{j}}|\langle \phi _{i}|\partial _{\varphi }\rho \left( \varphi \right) |\phi _{j}\rangle |^{2} =\sum _{i} \frac{(\partial _{\varphi }\lambda _{i})^{2}}{\lambda _{i}}+2 \sum _{i\ne j} \frac{(\lambda _{i}-\lambda _{j})^{2}}{\lambda _{i}+\lambda _{j}} |\langle \phi _{i}|\partial _{\varphi } \phi _{j} \rangle |^{2}, \end{aligned}$$

where $|\phi _{i}\rangle$ and $\lambda _{i}$ denote, respectively, the eigenvectors and eigenvalues of the density matrix $\rho \left( \varphi \right)$. In fact, the QFI is obtained by maximizing the Fisher information over all possible POVMs^4^, that is

7 $$\begin{aligned} F(\rho (\varphi ))=\max _{\{E_{x}\}} f(p(\varphi )), \end{aligned}$$

where $p(\varphi ) =\{p_{x}(\varphi )\}_{x}$ and $p_{x}(\varphi ) = \text {Tr}\{E_{x}\rho (\varphi )\}$. The fundamental relationship between the QFI and its corresponding quantum bound is expressed by the quantum Cramér-Rao bound^28^

8 $$\begin{aligned} \Delta \varphi _{QCR}=\sqrt{\dfrac{1}{F(\rho (\varphi ))}}, \end{aligned}$$

which sets the precision limit for quantum estimation of unknown parameter $\varphi$: $\Delta \varphi \ge \Delta \varphi _{QCR}$.

### Preliminaries: Hilbert–Schmidt speed (HSS)

We now report the definition of the Hilbert–Schmidt speed (HSS). Introducing the distance measure^30^

9 $$\begin{aligned} {[}d(p,q)]^{2}=\dfrac{1}{2}\sum \limits _{x}^{}|p_{x}-q_{x}|^{2}, \end{aligned}$$

where $p = \{p_{x}\}_{x}$ as well as $q = \{q_{x}\}_{x}$ are probability distributions, and subsequently considering the classical statistical speed

10 $$\begin{aligned} \text {s}[p(\varphi _{0})]=\dfrac{{\text{d}}}{{\text{d}}\varphi }d \big (p(\varphi _{0}+\varphi ),p(\varphi _{0})\big ), \end{aligned}$$

one can define a special kind of quantum statistical speed which is called HSS. Following the procedure discussed in the previous subsection for obtaining the corresponding quantum relations, one can determine the Hilbert–Schmidt distance^35^

11 $$\begin{aligned} \text {D}(\rho ,\sigma )\equiv \max _{\{E_{x}\}}\text {d}(\rho ,\sigma )=\sqrt{\frac{1}{2}\text {Tr}[(\rho -\sigma )^{2}]}, \end{aligned}$$

not requiring the diagonalization of the argument operator. The corresponding quantum statistical speed is just the HSS given by^30^

12 $$\begin{aligned} HSS (\rho (\varphi ))\equiv HSS_{\varphi } \equiv \text {S}[\rho (\varphi )]=\max _{\{E_{x}\}} \text {s}[p(\varphi )] =\sqrt{\frac{1}{2}\text {Tr}\bigg [\bigg (\dfrac{{\text {d}}\rho (\varphi )}{{\text {d}}\varphi }\bigg )^2\bigg ]}, \end{aligned}$$

which can be computed without diagonalizing $\text {d}\rho (\varphi )/\text {d}\varphi$. This is a significant advantage from a computational perspective.

### Quantum estimation through HSS

Because both QFI and HSS are quantum statistical speeds associated, respectively, with the Bures and Hilbert–Schmidt distances, it is reasonable to investigate how they can be related to each other. Based on analytical and numerical calculation, we find that an important relationship exists between them. We summarize our main result in the following.

**Main Result.** Suppose that we are given a pure initial state of an *n*-qubit quantum register such as

13 $$\begin{aligned} |\psi _{0}\rangle ={\mathcal {N}} \sum _{j=1}^d \text {e}^{i\varphi _{j}}c_{j}|j\rangle , \end{aligned}$$

where ${\mathcal {N}}={1}/{\sqrt{\sum _{j}|c_{j}|^{2}}}$ is the normalization factor and $\left\{ |j\rangle ,\ j=1,\ldots ,d \right\}$ denotes the computational basis of the collective Hilbert space of dimension *d*. Then, let this state be affected by an arbitrary quantum channel ${\mathcal {E}}_{t}$ giving the output state $\rho _{t} ={\mathcal {E}}_{t}(|\psi _{0}\rangle \langle \psi _{0} |)$. Under these conditions, we find that $HSS_{\varphi _{j}}\equiv HSS (\rho _{t}(\varphi _{j}))$ and $F_{\varphi _{j}}\equiv F (\rho _{t}(\varphi _{j}))$, computed with respect to phase parameter $\varphi _{j}$ encoded into the input state of Eq. (13), exhibit the same qualitative dynamics, that is:

(i) if $HSS_{\varphi _{j}} \ne 0$, one has ${\text {sgn}}\left( \frac{\text {d}HSS_{\varphi _{j}}}{\text {d}t}\right) ={\text {sgn}} \left( \frac{\text {d}F_{\varphi _{j}}}{\text {d}t}\right)$, where ${\text {sgn}}(x)$ indicates the sign of the argument *x*.
(ii) $$HSS_{\varphi _{j}}=0 \Leftrightarrow F_{\varphi _{j}}=0$$.

Notice that the relation (i) above immediately implies that maxima and minima times of QFI and HSS coincide. Therefore, by investigating the HSS dynamics we can faithfully detect the instants when the optimal phase estimation is achieved.

The sanity check of these relationships is performed by presenting various physical examples in the following subsections. It should be noted that using the general hierarchy between the HSS and QFI discussed in the context of quantum statistical speeds^30^, one can show that $0 \leqslant HSS_{\varphi _{j}} \leqslant \sqrt{F_{\varphi _{j}}}$, hence $F_{\varphi _{j}} = 0$ leads to $HSS_{\varphi _{j}} = 0$. However, the reverse (i.e., detecting the QFI zeros through the HSS zeros, which is the study of this paper) cannot be extracted from the above general inequality. Our subsequent analysis just confirms that if HSS vanishes then QFI also vanishes, leading to our main result. We also point out that, in the following, the special choice of the initial state of the system is chosen in order to simplify the analytical expressions of the QFI and the HSS. It is emphasized that our main result is independent of which one of the phase parameters $\varphi _{j}$ appearing in Eq. (13) is estimated. The results along the manuscript are general and hold for any other choices of the initial state where a relative phase parameter appears.

### One-qubit system

**Figure 1:**
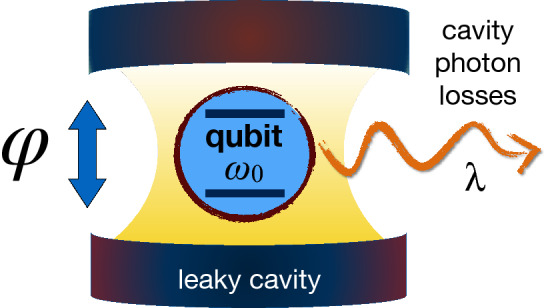
Sketch of a single qubit of transition frequency $\omega _0$ embedded in a dissipative zero-temperature cavity, with spectral width $\lambda$ (rate of photon losses). The qubit is initially prepared in a given superposition of its two states, with a relative phase parameter $\varphi$. This figure was created using Keynote, version 10.3.9 (7029.9.8), URL: https://apps.apple.com/it/app/keynote/id409183694?mt=12.

First we focus on the paradigmatic one-qubit system where the qubit interacts with a dissipative reservoir (cavity), as depicted in Fig. 1, through the Hamiltonian ($\hbar =1$)^36^

14 $$\begin{aligned} H=\omega _{0}~\sigma _{+}\sigma _{-}+\sum \limits _{k}^{}\omega _{k}b^{\dagger }_{k}b_{k} +(\sigma _{+}B+\sigma _{-}B^{\dagger }), \end{aligned}$$

where $\omega _{0}$ is the transition frequency of the qubit, $\sigma _{\pm }$ represent the system raising and lowering operators, $\omega _{k}$ is the frequency of the *k*-th field mode of the reservoir, $b_{k}$ ($b^{\dagger }_{k}$) denotes the *k*-mode annihilation (creation) operator, and $B=\sum _{k}^{}g_{k}b_{k}$ with $g_{k}$ being the coupling constant with the *k*-th mode. The cavity is initially in the vacuum state and uncorrelated from the qubit. Consider then the qubit prepared in the initial state

15 $$\begin{aligned} |\psi _{0}\rangle =\text {cos}\left( \frac{\theta }{2}\right) |1\rangle + \text {e}^{i\varphi }\text {sin}\left( \frac{\theta }{2}\right) |0\rangle , \end{aligned}$$

where the parameter $\varphi$ is the one to be estimated, while $\theta$ just fixes the probability amplitudes in the quantum superposition.

In the limit of a continuum of reservoir modes $\sum \nolimits _{k}^{}|g_{k}|^{2}\Rightarrow \int d\omega J(\omega )\delta (\omega _{k}-\omega )$, where $J(\omega )$ denotes the reservoir spectral density^36,37^. Restricting ourselves to the case of a single excitation in the atom-cavity system, we find that the cavity mode can be eliminated in favour of an effective spectral density of the following form^36^

16 $$\begin{aligned} J(\omega )=\dfrac{1}{2\pi }\dfrac{\gamma _{0} \lambda ^{2}}{(\omega _{0}-\omega )^{2}+\lambda ^{2}}. \end{aligned}$$

where parameter $\lambda$, connected to the reservoir correlation time $\tau _{c}$ by $\tau _{c} \approx 1/\lambda$, represents the spectral width for the qubit-reservoir coupling. Moreover, the decay rate $\gamma _{0}$ is related to the system (qubit) relaxation time scale $\tau _{r}$, over which the state of the system changes, by means of $\tau _{r} =1/\gamma _{0}$.

From the Hamiltonian of Eq. (14), at zero temperature and in the strong-coupling regime with the above Lorentzian spectral density for the cavity modes, one finds that the dynamics of the qubit in the basis $\{|1\rangle ,|0\rangle \}$ is described by the evolved reduced density matrix^36,38^

17 $$\begin{aligned} \rho ^{S}(t)=\left( \begin{array}{cc} \dfrac{P_{t} }{2} \left( \cos \left( \theta \right) +1 \right) &{}\dfrac{\sqrt{P_{t} }}{2}{{\text {e}}^{-i\varphi }}\sin \left( \theta \right) \\ \dfrac{\sqrt{P_{t} }}{2}{{\text {e}}^{i\varphi }} \sin \left( \theta \right) &{}1-\dfrac{P_{t} }{2} \left( \cos \left( \theta \right) +1 \right) \end{array} \right) , \end{aligned}$$

where the coherence characteristic function *P*(*t*) is

18 $$\begin{aligned} P(t)=\text {e}^{-\lambda t}\left[ \cos (\Gamma t/2)+(\lambda /\Gamma )\sin (\Gamma t/2)\right] ^{2}, \end{aligned}$$

with $$\Gamma =\sqrt{2\gamma _{0}\lambda -\lambda ^{2}}$$.

Inserting the time-dependent density matrix of Eq. (17) into Eqs. (6) and (12), we find that the QFI and HSS associated with an initial phase $\varphi$ are, respectively, given by

19 $$\begin{aligned} F_{\varphi }(t)=P_{t} \sin ^{2} \left( \theta \right) , \quad HSS_{\varphi }(t)=\dfrac{\sqrt{P_{t}}}{2}\sin \left( \theta \right) , \end{aligned}$$

leading to the relations

20 $$\begin{aligned} F_{\varphi }=4(HSS_{\varphi })^{2}\Longrightarrow \dfrac{\text {d}F_{\varphi }}{\text {d}t} =(8~HSS_{\varphi })\dfrac{\text {d}HSS_{\varphi }}{\text {d}t}. \end{aligned}$$

Accordingly, we see that when the HSS vanishes, the QFI also equals zero. Moreover, at all times when $HSS_{\varphi } \ne 0$, the signs of ${\text {d}F_{\varphi }}/{\text {d}t}$ and ${\text {d}HSS_{\varphi }}/{\text {d}t}$ coincide (HSS is a nonnegative quantity) and hence they exhibit the same qualitative dynamics. In particular, the times when the optimal estimation is achieved, i.e., ${\text {d}F_{\varphi }}/{\text {d}t} =0$ (maximum points of the QFI), can be easily detected by looking at the HSS dynamics.

### Two-qubit systems

The validity of our main result is here discussed for two-qubit systems in three different scenarios: local coupling to independent environments, coupling to a common environment, teleportation of entanglement between the two qubits.

#### Coupling to independent environments

**Figure 2:**
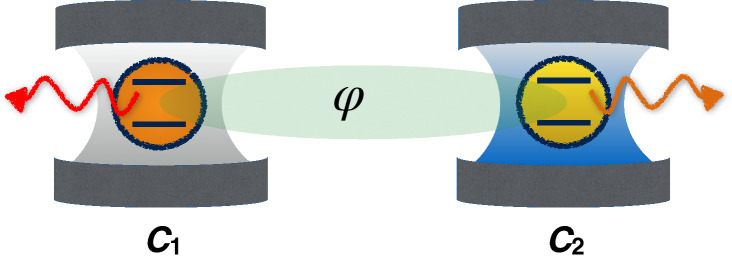
Sketch of two separated identical qubits each one interacting with its own zero-temperature dissipative cavity $C_i$ ($i=1,2$). The initial state of the two qubits is such that a relative phase parameter $\varphi$ appears between two-qubit basis states. This figure was created using Keynote, version 10.3.9 (7029.9.8), URL: https://apps.apple.com/it/app/keynote/id409183694?mt=12.

We consider a composite quantum system which consists of two separated identical qubits independently interacting with their own zero-temperature dissipative reservoir, as illustrated in Fig. 2. The two local qubit-reservoir interactions are assumed to be the same, for the sake of simplicity and without losing any generality. The two cavities (reservoirs), each exhibiting a Lorentzian spectral density, are initially in the vacuum state and uncorrelated from the two-qubit system. Knowing the evolved density matrix of the single qubit discussed in previous subsection, one can easily obtain the density matrix evolution of the two independent qubits^38–40^. In the computational basis $\{|11\rangle ,|10\rangle ,|01\rangle ,|00\rangle \}$, we take the two qubits initially prepared in the state

21 $$\begin{aligned} |\psi _{0}\rangle =\frac{1}{\sqrt{3}}(\text {e}^{i\varphi }|10\rangle +|01\rangle +|00\rangle ), \end{aligned}$$

with the phase parameter $\varphi$, giving rise to the evolved reduced density matrix

22 $$\begin{aligned} \rho ^{S}(t)=\left( \begin{array}{cccc} 0 &{} 0 &{} 0 &{} 0 \\ 0 &{} \dfrac{P_{t}}{3} &{} \dfrac{P_{t}}{3} e^{i \varphi } &{} \dfrac{\sqrt{P_{t}}}{3} e^{i \varphi } \\ 0 &{} \dfrac{P_{t}}{3} e^{-i \varphi } &{} \dfrac{P_{t}}{3} &{} \dfrac{\sqrt{P_{t}}}{3} \\ 0 &{} \dfrac{\sqrt{P_{t}} }{3} e^{-i \varphi } &{} \dfrac{\sqrt{P_{t}}}{3} &{} 1-\dfrac{2 P_{t}}{3} \\ \end{array} \right) , \end{aligned}$$

where $P_{t}\in [0,1]$ is the coherence characteristic function of Eq. (18).

Computing the QFI of Eq. (6) and the HSS of Eq. (12) with respect to the phase parameter $\varphi$, one promptly gets, respectively, the expressions

23 $$\begin{aligned} F_{\varphi }(t)=\dfrac{8}{9}P_{t},\quad HSS_{\varphi }(t)=\frac{1}{3} \sqrt{P_{t} (P_{t}+1)}, \end{aligned}$$

leading to

24 $$\begin{aligned} F_{\varphi }=\dfrac{4}{9}(\,\sqrt{1+36\,{{ HSS^{2}_{\varphi }}}}-1) \Rightarrow \dfrac{\text {d}F_{\varphi }}{\text {d}t} = {\frac{{ 16~ HSS_{\varphi }}}{\sqrt{1+36\,{{ HSS^{2}_{\varphi }}}}}} \dfrac{\text {d}HSS_{\varphi }}{\text {d}t}. \end{aligned}$$

The expressions above show once again our main result. In fact, assuming that $HSS_{\varphi _{j}} \ne 0$, we have that the two time derivatives ${\text {d}HSS_{\varphi _{j}}}/{\text {d}t})$ and ${\text {d}F_{\varphi _{j}}}/{\text {d}t})$ have the same sign. In addition, at times when $HSS_{\varphi _{j}}=0$, the QFI also vanishes and hence no information can be extracted from the system. Similar expressions would be obtained for different choices of the two-qubit initial state.

#### Coupling to a common environment

**Figure 3:**
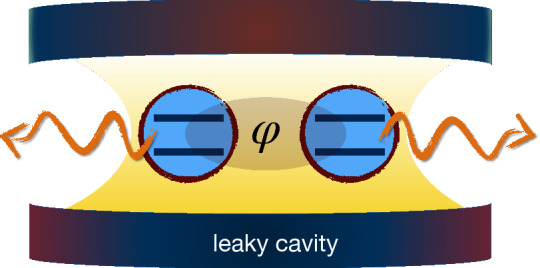
Illustration of two separated qubits both interacting with a zero-temperature common dissipative cavity. The initial state of the qubits is such that a relative phase parameter $\varphi$ appears between the two-qubit basis states. This figure was created using Keynote, version 10.3.9 (7029.9.8), URL: https://apps.apple.com/it/app/keynote/id409183694?mt=12.

We study two separated qubits interacting with a common zero-temperature bosonic reservoir, as depicted in Fig. 3. In such a case, the interaction between the two qubits is not direct but mediated by the common quantum reservoir. The total Hamiltonian of the system plus the reservoir is written as $H=H_{0} +H_{\text {int}}$, with^41^

25 $$\begin{aligned} H_{0} &= \omega _{1}~\sigma ^{(1)}_{+}\sigma ^{(1)}_{-}+\omega _{2}~\sigma ^{(2)}_{+} \sigma ^{(2)}_{-}+\sum \limits _{k}^{}\omega _{k}b^{\dagger }_{k}b_{k},\quad \text {(unperturbed Hamiltonian)}\nonumber \\ H_{\text{int}} &= (\alpha _{1}\sigma ^{(1)}_{+}+\alpha _{2}\sigma ^{(2)}_{+})B +(\alpha _{1}\sigma ^{(1)}_{-}+\alpha _{2}\sigma ^{(2)}_{-})B^{\dagger },\quad \text {(interaction Hamiltonian)} \end{aligned}$$

where $\sigma ^{(j)}_{\pm }$ and $\omega _{j}$ denote, respectively, inversion operator and transition frequency of the *j*-th qubit ($j=1,2$), $b^{\dagger }_{k}$ ($b_{k}$) represents the *k*-mode creation (annihilation) operator of quanta (photons) of the environment, and $B=\sum _{k}^{}g_{k}b_{k}$ in which $g_{k}$ is the coupling constant with the *k*-th mode. Moreover, the interaction of the *j*-th qubit with the reservoir is quantified by the dimensionless constant $\alpha _{j}$ depending on the value of the cavity field at the qubit position, which can be effectively controlled by means of dc Stark shifts tuning the atomic transition in and out of resonance. We investigate the case when the two atomic qubits interact resonantly with the reservoir described by a Lorentzian spectral density and they have the same transition frequency, i.e., $\omega _{1} =\omega _{2} = \omega _{0}$.

It is useful to introduce a collective coupling constant $\alpha _{T}=\sqrt{\alpha ^{2}_{1}+\alpha ^{2}_{2}}$, the relative strengths $r_{j}=\alpha _{j}/\alpha _{T}$ such that $r_{1}^{2}+r_{2}^{2}=1$, and mutually orthogonal quantum states

26 $$\begin{aligned} | \psi _{ +}\rangle =r_{1}| 10\rangle +r_{2}| 01\rangle ,\quad | \psi _{ -}\rangle =r_{2}| 10\rangle -r_{1}| 01\rangle . \end{aligned}$$

The cavity is initially in its vacuum state $| \mathbf{0} \rangle =\bigotimes _{k} | 0_{k}\rangle$, uncorrelated from the two-qubit system. With these definitions, for an initial two-qubit state of the form

27 $$\begin{aligned} | \psi _{ 0}\rangle =\dfrac{1}{\sqrt{2}}(| 10\rangle +\text {e}^{i\varphi }| 01\rangle ), \end{aligned}$$

one finds that the evolved reduced density matrix of the two-qubit system in the computational basis $\{|11\rangle ,|10\rangle ,|01\rangle ,|00\rangle \}$ is given by^41^

28 $$\begin{aligned} \rho (t)=\left( \begin{array}{cccc} 0&{} 0&{}0&{}0 \\ 0 &{}|c_{1}(t)|^{2}&{}c_{1}(t)c^{*}_{2}(t)&{}0 \\ 0 &{}c^{*}_{1}(t)c_{2}(t) &{}|c_{2}(t)|^{2}&{}0 \\ 0 &{} 0&{}0&{}1-|c_{1}(t)|^{2}-|c_{2}(t)|^{2} \\ \end{array} \right) , \end{aligned}$$

where, defining $\beta _{\pm }= \langle \psi _{ \pm } | \psi _{ 0}\rangle$, one has

29 $$\begin{aligned} c_{1}(t)=r_{2}\beta _{-}+r_{1}{\mathcal {F}}_{t}\beta _{+},\quad c_{2}(t)=-r_{1}\beta _{-}+r_{2}{\mathcal {F}}_{t}\beta _{+}. \end{aligned}$$

Moreover, introducing the dimensionless quantities $\tau =\lambda t$ and $R={\mathcal {R}}/\lambda$, where $1/\lambda$ is the reservoir correlation time and ${\mathcal {R}}$ denotes the vacuum Rabi frequency, one obtains

30 $$\begin{aligned} {\mathcal {F}}_{\tau }=\text {e}^{-\tau /2}\left[ \cosh \left( \frac{\tau }{2} \sqrt{1-4R^{2}}\right) +\dfrac{1}{\sqrt{1-4R^{2}}}\sinh \left( \frac{\tau }{2} \sqrt{1-4R^{2}}\right) \right] . \end{aligned}$$

The above equations allow us to calculate the relevant quantities for our study, that is QFI and HSS. The QFI can be computed analytically but its expression is too cumbersome to be presented here. On the other hand, we find that the HSS is given by

31 $$\begin{aligned} HSS_{\varphi }(t)=\dfrac{1}{2} \left( {r_{{1}}}^{2}+{r_{{2}}}^{2} \right) \sqrt{{{\mathcal {F}}_{t}}^{2} {r_{{1}}}^{4}-2\, \left( {{\mathcal {F}}_{t}}^{2}-1 \right) ^{2}{r_{{1}}}^{2}{r_ {{2}}}^{2}\cos \left( 2\,\varphi \right) +2\, \left( {{\mathcal {F}}_{t}}^{4}-{ {\mathcal {F}}_{t}}^{2}+1 \right) {r_{{1}}}^{2}{r_{{2}}}^{2}+{{\mathcal {F}}_{t}}^{2}{r_{{ 2}}}^{4}}. \end{aligned}$$

**Figure 4:**
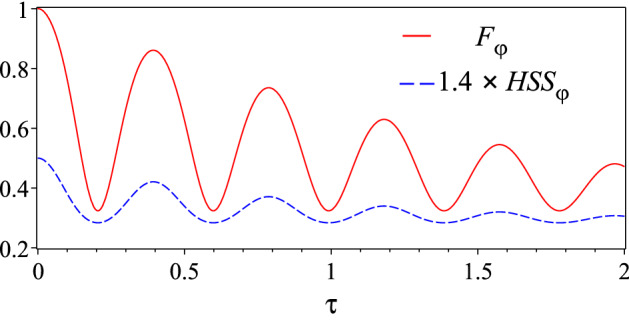
Dynamics of quantum Fisher information $F_{\varphi }(t)$ (red solid line) and Hilbert–Schmidt speed $HSS_{\varphi }(t)$ (amplified by 1.4 times for comparison, blue dashed line), as a function of the dimensionless time $\tau$ for the two-qubits system coupled to a common reservoir, with $r_{1}=0.3$ and $R=8$. Notice that $F_{\varphi }(t)$ approximately oscillates between 0.3 and 1 due to the special choice of the initial parameters.

The dynamical comparison between the two figures of merit is then performed by plots. In particular, we show in Fig. 4 that both the QFI and HSS dynamics simultaneously exhibit an oscillatory behavior such that their maximum and minimum points exactly coincide. This plot qualitatively verifies our result that the HSS can detect exactly the times at which the best phase estimation occurs (maximum of the QFI). Therefore, also for this system, the HSS, similarly to QFI, can be used as a distinguishability metric on the space of quantum states which quantifies the maximum amount of information on an unknown phase parameter attainable by a given probe state.

#### Two-qubit entanglement teleportation

We now study the relationship between QFI and HSS within the scenario of noisy two-qubit teleportation, where a two-qubit entangled state is to be teleported from a place to another^42,43^. Let us first describe the noisy two-qubit system to this aim, which shall constitute the resource channel for the teleportation.

One of the most important noise models used in the low-temperature regime is described by a spin environment^44,45^. In particular, in order to achieve suitable operations in experiments performed to study macroscopic quantum coherence and decoherence, one requires temperatures close to absolute zero. Here we consider a two-qubit system interacting with an external environment composed of *N* spins. The general Hamiltonian is written as $H=H_{S}+H_{E}+H_{I}$ where the system, environment and interaction Hamiltonians are given, respectively, by^44,45^

32 $$\begin{aligned} H_{S}=\dfrac{\hbar \Omega _{1}}{2}\sigma _{z}^{1}+\dfrac{\hbar \Omega _{2}}{2}\sigma _{z}^{2}+\gamma \sigma _{z}^{1}\sigma _{z}^{2},\quad H_{E}=\sum \limits _{i=1}^{N} h_{i} \sigma _{xi},\quad H_{I}=\sigma _{z}^{1}\otimes \sum \limits _{n=1}^{N}\varepsilon _{i}\sigma _{zi}+\sigma _{z}^{2}\otimes \sum \limits _{n=1}^{N}\lambda _{i}\sigma _{zi}, \end{aligned}$$

where $\Omega _{i}$ and $\gamma$ denote, respectively, the characteristic frequency of the *i*-th qubit and the coupling strength between the two spin qubits. Moreover, $h_{i}$ denotes the tunneling matrix element for the *i*-th environmental spin, while $\varepsilon _{i}$ ($\lambda _{i}$) represents the coupling between qubit 1 (qubit 2) and the spins of the environment.

Initializing the two-qubit system in an extended Werner-like state^40^

33 $$\begin{aligned} \rho (0)=\frac{1-r}{4}{\mathbb {I}}_4+r|\vartheta \rangle \langle \vartheta |, \end{aligned}$$

where $r \in (0,1]$ quantifies the degree of purity of the state, ${\mathbb {I}}_4$ is the $4\times 4$ identity matrix and

34 $$\begin{aligned} | \vartheta \rangle =\sqrt{1-p}|00\rangle +\sqrt{p}|11\rangle , \quad (0\le p \le 1), \end{aligned}$$

one finds that the evolved reduced density matrix in the two-qubit computational basis $\{|00\rangle ,|01\rangle ,|10\rangle ,|11\rangle \}$ is given by^45^

35 $$\begin{aligned} \rho (t)=\left( \begin{array}{cccc} \dfrac{1-r}{4}+r(1-p)&{} 0&{}0&{}r\sqrt{p(1-p)}e^{-i(\Omega _{1}+\Omega _{2})t} Q(t) \\ 0 &{}\dfrac{1-r}{4}&{}0&{}0 \\ 0 &{}0 &{}\dfrac{1-r}{4}&{}0 \\ r\sqrt{p(1-p)}e^{i(\Omega _{1}+\Omega _{2})t} Q(t) &{} 0&{}0&{}\dfrac{1-r}{4}+rp \\ \end{array} \right) , \end{aligned}$$

where the decoherence factor *Q*(*t*) is

36 $$\begin{aligned} Q(t)=\prod \limits _{i=1}^{N}\left\lgroup 1-\left[ \dfrac{2(\varepsilon _{i}+\lambda _{i})^{2}}{h_{i}^{2}+(\varepsilon _{i} +\lambda _{i})^{2}}\right] \text {sin}^{2}(t\sqrt{h_{i}^{2}+(\varepsilon _{i}+\lambda _{i})^{2}})\right\rgroup . \end{aligned}$$

**Figure 5:**
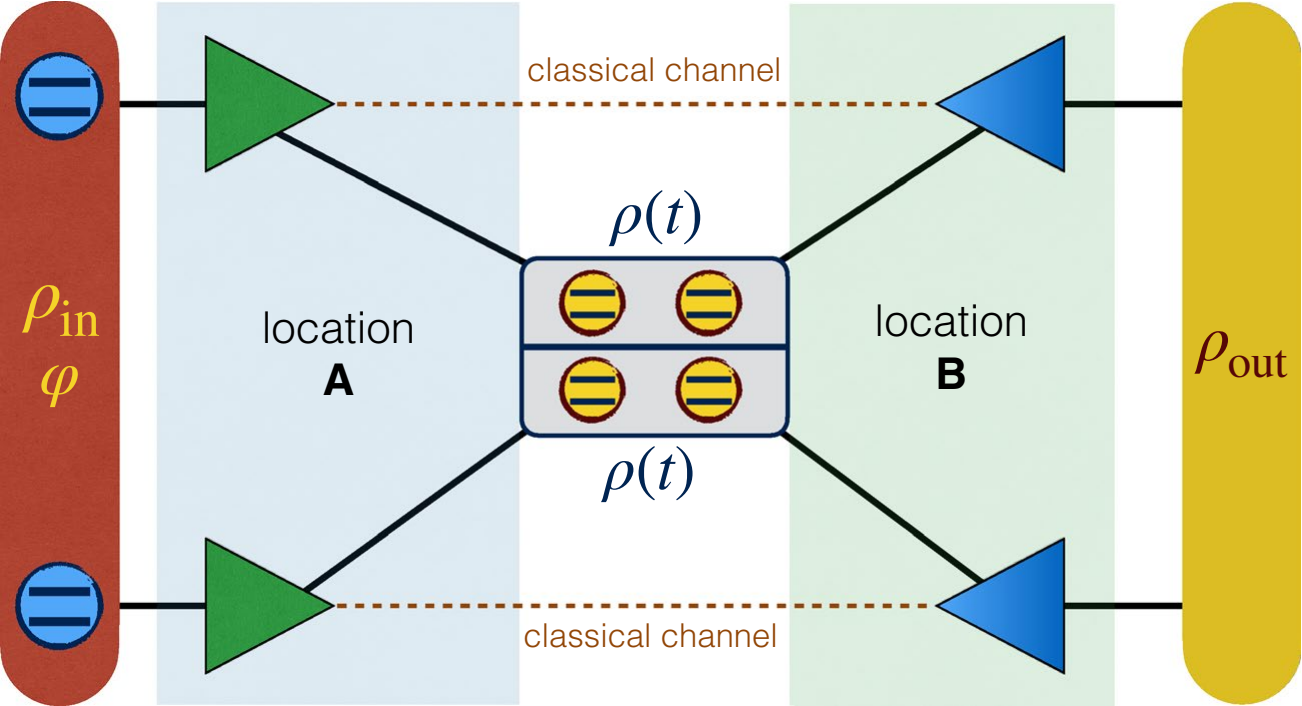
Sketch of the noisy two-qubit teleportation protocol. Two copies of the evolved noisy two-qubit state $\rho (t)$ are employed as resource channel for the protocol, shared between the two locations A and B. The input state $\rho _{\text {in}}$ to be teleported, containing information about the phase parameter $\varphi$, is manipulated in location A. After the protocol, the state $\rho _{\text {out}}$ is teleported at the location B encoded in the two qubits coming from the resource state^42^. This figure was created using Keynote, version 10.3.9 (7029.9.8), URL: https://apps.apple.com/it/app/keynote/id409183694?mt=12.

Assuming that the two qubits are shared between Alice (location A) and Bob (location B), we use two copies of this system state as a resource channel for the teleportation of an unknown entangled state $\rho _{\text {in}}$ from A to B, according to a basic scheme^42^ illustrated in Fig. 5. It is useful to introduce the Bell states ${\mathcal {B}}_{i}$’s associated with the Pauli matrices $\sigma _{i}$’s by

37 $$\begin{aligned} {\mathcal {B}}_{i}=\left( \sigma _{0}\otimes \sigma _{i}\right) {\mathcal {B}}_{0} \left( \sigma _{0}\otimes \sigma _{i}\right) ,\quad (i=1,2,3), \end{aligned}$$

where $\sigma _{0}={\mathbb {I}}_2$ (${\mathbb {I}}_2$ being the $2\times 2$ identity matrix), $\sigma _{1}=\sigma _{x}$, $\sigma _{2}=\sigma _{y}$, $\sigma _{3}=\sigma _{z}$ and ${\mathcal {B}}_{0}=\frac{1}{2}\left( |00\rangle +|11\rangle \right) \left( \langle 00| +\langle 11|\right)$. Now, one can generalize the standard teleportation protocol^42^ and find that the output state of the two-qubit teleportation is^43^

38 $$\begin{aligned} \rho _{\text {out}}=\sum _{ij}p_{ij}\left( \sigma _{i}\otimes \sigma _{j}\right) \rho _{\text {in}}\left( \sigma _{i}\otimes \sigma _{j}\right) ,\quad (i,j=0,1,2,3), \end{aligned}$$

where $p_{ij}=\text {Tr}\left( {\mathcal {B}}_{i}\rho _{\text {res}} \right) \text {Tr}\left( {\mathcal {B}}_{j}\rho _{\text {res}} \right)$, $\rho _\text {res}$ being the resource state for the teleportation which is equal to the reduced density matrix of Eq. (35) in our model. Accordingly, for the input state $\rho _{\text {in}}=|\psi _{\text {in}}\rangle \langle \psi _{\text {in}}|$ with

39 $$\begin{aligned} |\psi _{\text{in}}\rangle ={\text {cos}}(\theta /2)|10\rangle +{\text {sin}}(\theta /2) {\text {e}}^{i\varphi }|01\rangle , \end{aligned}$$

where $\ 0\le \theta \le \pi ,\ 0\le \varphi \le 2\pi$, we find that the output state of the teleportation is given by

40 $$\begin{aligned} \rho _{\text{out}}(t)=\left[ \begin{array}{cccc} 4\,{R}^{2}+2\,Rr&{}0&{}0&{}0 \\ 0&{} \left( 4\,Rr+{r}^{2} \right) \sin ^{2} \left( \dfrac{\theta }{2} \right) +4\,{R}^{2}&{}2\,{e}^{i\varphi } \sin \left( \theta \right) {A^{2}(t)} \cos ^{2} \big ( \Omega _{{1}}+\Omega _{{2}} \big ) &{}0 \\ 0&{}2\,{e}^{-i\varphi } \sin \left( \theta \right) {A^{2}(t)} \cos ^{2} \big ( \Omega _{{1}}+\Omega _{{2}} \big ) &{} \left( 4\,Rr+{r}^{2} \right) \cos ^{2} \left( \dfrac{ \theta }{2} \right) +4\,{R}^{2} &{}0\\ 0&{}0&{}0&{}4\,{R}^{2}+2\,Rr\end{array} \right] , \end{aligned}$$

with $R={(1-r)}/{4}$ and $A(t)=r Q(t)\sqrt{ p(1-p)}$. Using this expression for the output state, we find that the QFI and HSS associated to the phase parameter $\varphi$ encoded into the input state $\rho _{\text{in}}$ sent into the teleportation channel are, respectively,

41 $$\begin{aligned} F_{\varphi }(t)=32\,{\frac{{A}^{4}(t) \cos ^{4} \big ( \Omega _{{1}}+\Omega _{{2}} \big ) \sin ^{2} \theta }{1+{r}^{2}}},\quad HSS_{\varphi }(t)=2\,{A}^{2}(t) \cos ^{2} \big ( \Omega _{{1}}+\Omega _{{2}} \big ) \sin \theta . \end{aligned}$$

From these expressions we straightforwardly obtain

42 $$\begin{aligned} F_{\varphi }={\frac{{{ 8~HSS^{2}_{\varphi }}}}{1+{r}^{2}}} \Longrightarrow \dfrac{\text {d}F_{\varphi }}{\text {d}t}={\frac{{{ 16~HSS_{\varphi }}}}{1+{r}^{2}}}\dfrac{\text {d}HSS_{\varphi }}{\text {d}t}, \end{aligned}$$

leading to our main result, i.e., the possibility of extracting the QFI dynamics through the HSS dynamics due to the fact that the two figures of merit have the same qualitative time behavior.

### General case: *n*-qubit system ($n\ge 3$)

Now the validity of the main result is investigated for high-dimensional systems in two scenarios: local coupling to independent environments and coupling to a common environment.

#### Coupling to independent environments

Here we analyze the case of an open quantum system made of *n* noninteracting qubits locally subject to their own reservoir, a typical configuration for quantum networking with quantum registers. To this aim, one has to choose the type of qubit and the local qubit-environment interaction.

We specifically consider the dynamics of a topological qubit realized by two Majorana modes which are generated at the endpoints of some nanowire with strong spin-orbit interaction, placed on top of an s-wave superconductor and driven by an external magnetic field **B** along the wire axis direction^46,47^. We also assume that each Majorana mode is coupled to the metallic nanowire via a tunnel junction in the way that the tunneling strength is controllable by an external gate voltage. The total Hamiltonian is written as

43 $$\begin{aligned} H=H_{S}+H_{E}+V \end{aligned}$$

where $H_{S}$ denotes the Hamiltonian of the topological qubit, *V* represents the system-environment interaction Hamiltonian, while $H_{E}$ is the environment Hamiltonian whose elementary constituents can be considered as electrons or holes. The decoherence which affects the topological qubit is modeled as a fermionic Ohmic-like environment described by spectral density $J(\omega ) \propto \omega ^{Q}$ with $Q\ge 0$. The Ohmic, super-Ohmic and sub-Ohmic environments are characterized by $Q = 1$, $Q > 1$ and $Q < 1$, respectively. Since these Majorana modes used as the topological qubit are zero-energy modes, we have $H_{S}=0$. Moreover, the interaction Hamiltonian *V*, constructed by the electron creation (annihilation) operators with Majorana modes $\gamma _{1}$ and $\gamma _{2}$, satisfies the properties

44 $$\begin{aligned} \gamma ^{\dagger }_{a}=\gamma _{a},\quad \{\gamma _{a},\gamma _{b}\}=2\delta _{ab}, \quad (a,b=1,2 ). \end{aligned}$$

Before turning on the interaction *V*, the two Majorana modes construct a topological (non-local) qubit with states $|0\rangle$ and $|1\rangle$ related to each other by

45 $$\begin{aligned} \frac{1}{2}(\gamma _{1}-\text {i}\gamma _{2})|0\rangle =|1\rangle ,\quad \frac{1}{2}(\gamma _{1}+\text {i}\gamma _{2})|1\rangle =|0\rangle , \end{aligned}$$

where $\gamma _{1}$, $\gamma _{2}$ are chosen to be represented by $\gamma _{1}=\sigma _{1}$, $\gamma _{2}=\sigma _{2}$, $\text {i}\gamma _{1}\gamma _{2}=\sigma _{3}$, in which $\sigma _{j}$’s are the usual Pauli matrices.

Let us now assume, as usual, that the state $\varrho ^{T}$ of the total qubit-environment system is uncorrelated initially: $\varrho ^{T}(0)=\varrho (0)\otimes \varrho _{E}$, where $\rho _{S}(0)$ and $\rho _{E}$ are the initial density matrices of the topological qubit and of its environment, respectively. Supposing that the initial state of the Majorana qubit is written as

46 $$\begin{aligned} \varrho (0)=\left( \begin{array}{cc} \varrho _{11}(0)&{}\varrho _{12}(0) \\ \varrho _{21}(0)&{}\varrho _{22}(0) \\ \end{array}\right) , \end{aligned}$$

one finds that the reduced density matrix of the topological qubit at time *t* can be obtained by a dynamical map $\Phi _{t}$ such that (for details, see Ref.^46^)

47 $$\begin{aligned} \varrho (t)=\Phi _{t}\big (\varrho (0)\big )= \frac{1}{2}\left( \begin{array}{cc} 1+(2\varrho _{11}(0)-1)\alpha ^{2}(t)&{}2\varrho _{12}(0)\alpha (t) \\ 2\varrho _{21}(0)\alpha (t)&{}1+(2\varrho _{22}(0)-1)\alpha ^{2}(t) \\ \end{array}\right) , \end{aligned}$$

where

48 $$\begin{aligned} \alpha (t)=\text {e}^{-2B^{2}|\beta |I_{Q}(t)},\quad \beta \equiv \dfrac{-4\pi }{\Gamma (Q+1)}\left( \dfrac{1}{\varGamma _{0}}\right) ^{Q+1},\quad I_{Q}(t)= \left\{ \begin{array}{rl} 2\varGamma _{0}^{Q-1} \Gamma (\frac{Q-1}{2})\bigg [1-\,_1F_1\big (\frac{Q-1}{2}; \frac{1}{2};-\frac{t^{2}\varGamma ^{2}_{0}}{4}\big )\bigg ], &{} Q\ne 1,\\ \frac{1}{2}t^{2}\varGamma ^{2}_{0} \,_2F_2\bigg (\{1,1\};\{3/2,2\};-\frac{t^{2}\varGamma ^{2}_{0}}{4}\bigg ), &{} Q=1, \end{array} \right. \end{aligned}$$

with $\varGamma _{0}$ indicating the high-frequency cutoff for the linear spectrum of the edge state, $\Gamma (z)$ representing the Gamma function, and ${}_pF_q$ being the *generalized hypergeometric function* . From the eigenvalues and eigenvectors of the *Choi matrix*^48^ of the map $\Phi _{t}$, we obtain the corresponding operator-sum representation $\varrho (t)=\sum _{i=1}^4 K_i(t) \varrho (0) K_i(t)^\dagger$ with Kraus operators $\{K_{i}(t)\}$ given by

49 $$\begin{aligned}&K_{1}(t)=\left( \begin{array}{cc} \frac{\alpha -1}{2} &{} 0 \\ 0 &{} \frac{1-\alpha }{2} \\ \end{array} \right) ,~K_{2}(t)=\left( \begin{array}{cc} \frac{\alpha +1}{2} &{} 0 \\ 0 &{} \frac{\alpha +1}{2} \\ \end{array} \right) ,\nonumber \\&K_{3}(t)=\left( \begin{array}{cc} 0 &{} \frac{\sqrt{1-\alpha ^2}}{\sqrt{2}} \\ 0 &{} 0 \\ \end{array} \right) ,~K_{4}(t)=\left( \begin{array}{cc} 0 &{} 0 \\ \frac{\sqrt{1-\alpha ^2}}{\sqrt{2}} &{} 0 \\ \end{array} \right) . \end{aligned}$$

**Figure 6:**
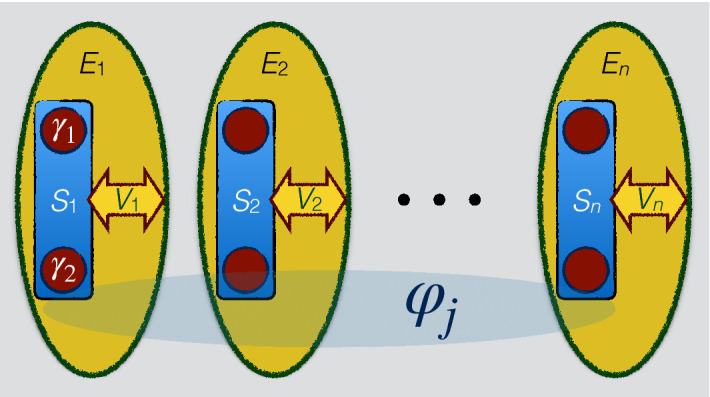
Illustration of a system of *n* noninteracting topological qubits $S_i$ ($i=1,2,\ldots ,n$), each one built by two Majorana modes $\gamma _{2i-1}$ and $\gamma _{2i}$ (only $\gamma _1$, $\gamma _2$ are explicitly indicated for simplicity), locally interacting by $V_i$ to the environment $E_i$ (constituted by electrons and holes). The *n* qubits are initially prepared in a state containing relative phase parameters $\varphi _j$ between the computational basis states. This figure was created using Keynote, version 10.3.9 (7029.9.8), URL: https://apps.apple.com/it/app/keynote/id409183694?mt=12.

In the following, we take a system formed by *n* noninteracting topological qubits $S_i$ (each one defined by two Majorana modes as above) such that each qubit locally interacts with the environment $E_i$ described above by the interaction $V_i$ ($i=1,2,\ldots ,n$), as displayed in Fig. 6. Note that the effects of the environment on each of the qubits can be canceled by setting the corresponding external magnetic field $\mathbf{B}$ to zero. With this in mind, we focus on the scenarios in which *m* of the qubits are affected by the noise, while the remaining ones ($n-m$) are noiseless. Since the environments are independent in our model, the Kraus operators are just tensor products of Kraus operators acting on each of the qubits, noting that the Kraus operators of the noiseless qubits are set to identity operator^38,49^.

We start by considering a three-qubit system $(n=3)$ with $m=3$, initially prepared in the W-like state^50^

50 $$\begin{aligned} |W\rangle _{3}=\dfrac{1}{\sqrt{3}}\left( \text {e}^{i\varphi _{1}}|100\rangle +|010\rangle +\text {e}^{i\varphi _{2}}|001\rangle \right) , \end{aligned}$$

where two phase parameters $\varphi _{1}$, $\varphi _2$ appear. We find that the QFI and HSS associated with phase parameter $\varphi _{1}$ is obtained as

51 $$\begin{aligned} F_{\varphi _{1}}(t)=\frac{2}{9} \left( 5-\frac{2}{\alpha ^2(t)+1}\right) ,~HSS_{\varphi _{1}}(t)=\frac{\alpha ^2(t)+1}{3 \sqrt{2}}. \end{aligned}$$

It is easily found that

52 $$\begin{aligned} F_{\varphi _{1}}(t)={\frac{10}{9}}-{\frac{2\,\sqrt{2}}{27\,{ HSS_{\varphi _{1}}}}}(t)\Rightarrow \dfrac{\text {d}F_{\varphi _{1}}}{\text {d}t}={\frac{2\,\sqrt{2}}{27\,{{ (HSS_{\varphi _{1}}}})^{2}}} \dfrac{\text {d}HSS_{\varphi _{1}}}{\text {d}t}. \end{aligned}$$

**Figure 7:**
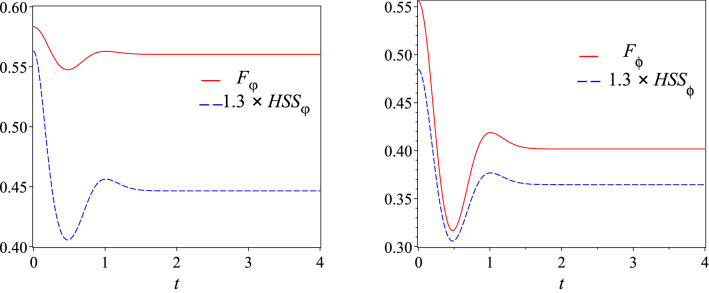
Dynamics of quantum Fisher information *F*(*t*) (red solid line) and Hilbert–Schmidt speed *HSS*(*t*) (amplified by 1.3 times for comparison, blue dashed line) which are computed with the same phase parameter as a function of time *t* for the (**a**) four-qubit system prepared in initial state (55) and (**b**) six-qubit system prepared in initial state (56) described in Fig. 6 when one of the qubits is affected by the noise.

As clear from Eq. (51), the HSS is always nonzero and we conclude that the QFI dynamics can be completely determined by analyzing the HSS dynamics: our main result is once again proved. Now we compute the measures associated with the phase parameter $\varphi _{2}$, leading to the expressions

53 $$\begin{aligned} F_{\varphi _{2}}(t)=\frac{16 \alpha ^2(t)}{9 \alpha ^2(t)+9},\quad HSS_{\varphi _{2}}(t)=\frac{\sqrt{2} \alpha (t) }{3}, \end{aligned}$$

from which we obtain

54 $$\begin{aligned} F_{\varphi _{2}}(t)=\dfrac{16~ \bigg (HSS_{\varphi _{2}}(t)\bigg )^{2}}{9~ \bigg (HSS_{\varphi _{2}}(t)\bigg )^{2}+2} \Rightarrow \dfrac{\text {d}F_{\varphi _{2}}}{\text {d}t}= \,{\frac{{64~ HSS_{\varphi _{2}}}}{ \left[ 9\,(HSS_{\varphi _{2}}) ^{2}+2 \right] ^{2}}} \dfrac{\text {d}HSS_{\varphi _{2}}}{\text {d}t}, \end{aligned}$$

so that our main result is manifestly confirmed also in this case.

In Fig. 7, we numerically illustrate the same qualitative time behavior of the HSS and QFI for two different initial states: (a) a four-qubit system $(n=4)$ and (b) a six-qubit system $(n=6)$ (with $m=1$), initially prepared in the following W-like states

55 $$\begin{aligned} |W\rangle _{4}=\dfrac{1}{\sqrt{4}}\big (|1000\rangle +\text {e}^{i\varphi }|0100\rangle +|0010\rangle +|0001\rangle \big ), \end{aligned}$$

and

56 $$\begin{aligned} |W\rangle _{6}=\dfrac{1}{\sqrt{6}}\big (\text {e}^{i\varphi }|100000\rangle + \text {e}^{i\phi }|010000\rangle +|001000\rangle +|000100\rangle +|000010\rangle +|000001\rangle \big ), \end{aligned}$$

respectively.

As another example, we choose *n* and *m* arbitrary with the *n*-qubit register prepared in a Greenberger-Horne-Zeilinger (GHZ)-like state^50,51^ written as

57 $$\begin{aligned} |{\text {GHZ}}\rangle _{n}=\dfrac{1}{\sqrt{2}}\big (\text {e}^{i\varphi }|0\rangle ^{\otimes n}+|1\rangle ^{\otimes n}\big ), \end{aligned}$$

where the relevant phase parameter appears. Calculating the evolved state of the system, we find that the corresponding QFI and HSS are given, respectively, by

58 $$\begin{aligned} F^{GHZ}_{n,m}(\varphi )=F_{\varphi }=\bigg (\dfrac{2\alpha ^{2}}{1+\alpha ^{2}} \bigg )^{m},\quad HSS^{GHZ}_{n,m}(\varphi )=HSS_{\varphi }=\dfrac{\alpha ^{m}}{2}. \end{aligned}$$

Hence, we can write

59 $$\begin{aligned} F_{\varphi }= 2^{m+2}(HSS_{\varphi })^2 \left( 4^{1/m} (HSS_{\varphi })^{2/m}+1\right) ^{-m}\Longrightarrow \dfrac{\text {d}F_{\varphi }}{\text {d}t}= 2^{m+3}(HSS_{\varphi }) \left( 4^{1/m} (HSS_{\varphi })^{2/m}+1\right) ^{-m-1}\dfrac{\text {d}HSS_{\varphi }}{\text {d}t}, \end{aligned}$$

explicitly leading to our main result also in this general case of *n* qubits, as already confirmed by the other previous examples with a fixed number of qubits.

#### Coupling to a common environment: depolarizing channel

**Figure 8:**
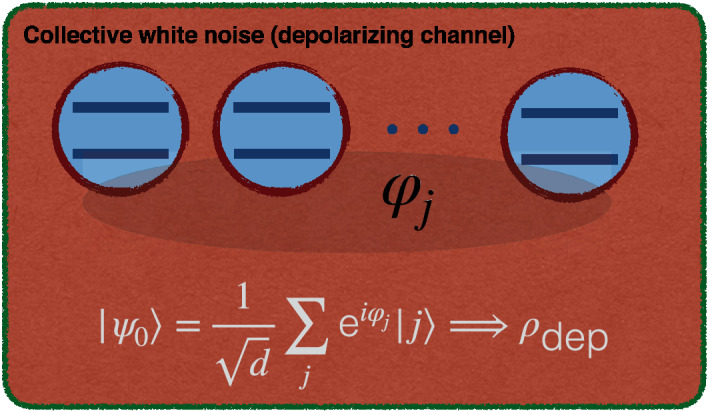
Illustration of an *n*-qubit register collectively subject to white noise under the action of a common depolarizing channel. This channel turns an initial pure state $|\psi _{0}\rangle$ into the mixture $\rho _{\text {dep}}=\eta |\psi _{0}\rangle \langle \psi _{0}| +\frac{1-\eta }{d}{\mathbb {I}}_{d\times d}$. This figure was created using Keynote, version 10.3.9 (7029.9.8), URL: https://apps.apple.com/it/app/keynote/id409183694?mt=12.

Now we investigate the phase estimation problem for a natural parametrization of arbitrary pure states affected by the (isotropic) depolarizing channel^49^ given by the map

60 $$\begin{aligned} |\psi _{0}\rangle \Longrightarrow \rho _{\text {dep}}=\eta |\psi _{0}\rangle \langle \psi _{0}| +\frac{1-\eta }{d}{\mathbb {I}}_{d\times d}, \end{aligned}$$

in which $\eta$ and *d* denote the * reliability* of the channel and dimension of the Hilbert space, respectively. This form of collective channel, mixing the initial *n*-qubit pure state with white noise and illustrated in Fig. 8, plays an important role in various quantum information tasks, such as quantum cloning machines^52^, NMR quantum computing^53^, entanglement optimization^54^, quantum error correction^55^, and quantum repeaters^56^. We focus on the following parametrization of input pure states

61 $$\begin{aligned} |\psi _{0}\rangle =\frac{1}{\sqrt{d}} \sum _{j} \text {e}^{i\varphi _{j}}|j\rangle . \end{aligned}$$

After lengthy calculation one can find that the general expression of QFI associated with any parameter $\varphi _{j}$ is given by^57^

62 $$\begin{aligned} F_{\varphi _{j}}=\dfrac{4(d-1)\eta ^{2}}{2d+d(d-2)\eta }. \end{aligned}$$

Moreover, we obtain that the HSS corresponding to the parameter $\varphi _{j}$ can be represented as

63 $$\begin{aligned} HSS_{\varphi _{j}}=\frac{\sqrt{d-1}}{d}\eta , \end{aligned}$$

leading to the following relationship between QFI and HSS:

64 $$\begin{aligned} F_{\varphi _{j}}=\,{\frac{4d\sqrt{d-1}{{ HSS}}^{2}_{\varphi _{j}}}{({d}^{2}-2d){ HSS}_{\varphi _{j}}+2\,\sqrt{d-1}}} \Longrightarrow \dfrac{\text {d}F_{\varphi _{j}}}{\text {d}t}= 4\,{\frac{ \left( 4\,\sqrt{d-1}+d \left( d-2 \right) {HSS_{\varphi _{j}}} \right) d\sqrt{d-1}{HSS_{\varphi _{j}}}}{ \left( 2\,\sqrt{d-1}+d \left( d-2 \right) {HSS_{\varphi _{j}}} \right) ^{2}}} \dfrac{\text {d}HSS_{\varphi _{j}}}{\text {d}t}. \end{aligned}$$

Because $d=2^{n} \ge 2$, our main result can be immediately confirmed. Combining this result with the fact that $\dfrac{\text {d}HSS_{\varphi _{j}}}{\text {d}t}=\big (\sqrt{d-1}/d\big )\dfrac{\text {d}\eta }{\text {d}t}$ (see Eq. (63)), we see that when the reliability parameter of the depolarizing channel oscillates with time, an indication of non-Markovianity^58^, the oscillations are in turn detected easily by the HSS.

#### Coupling to a common environment: dephasing channel

**Figure 9:**
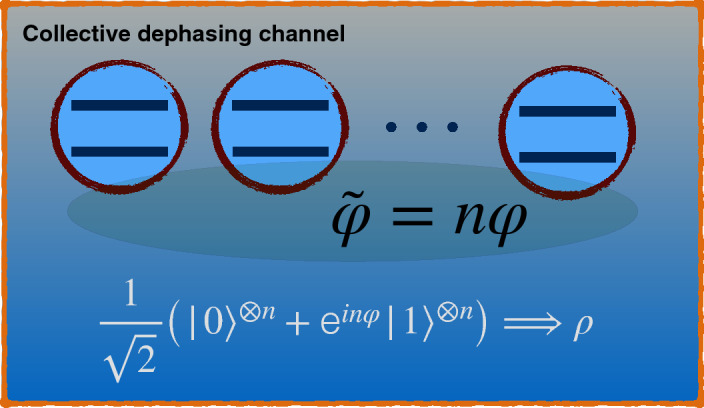
Illustration of an *n*-qubit register subject to the action of a common dephasing channel. This channel turns an initial pure GHZ state $|{\widetilde{GHZ}}\rangle _{n}=\frac{1}{\sqrt{2}}\big (|0\rangle ^{\otimes n}+\text {e}^{in\varphi }|1\rangle ^{\otimes n}\big )$ into a mixed state $\rho$. This figure was created using Keynote, version 10.3.9 (7029.9.8), URL: https://apps.apple.com/it/app/keynote/id409183694?mt=12.

Here, the validity of our main result is checked for the dynamics of an *n*-qubit system with total angular momentum $j=n/2$ in the presence of collisional dephasing, as depicted in Fig. 9. Using a unitary evolution, we can prepare the probes in the following GHZ-like initial state

65 $$\begin{aligned} |{\widetilde{GHZ}}\rangle _{n}=\dfrac{1}{\sqrt{2}}\big (|0\rangle ^{\otimes n}+\text {e}^{in\varphi }|1\rangle ^{\otimes n}\big ) \equiv \dfrac{1}{\sqrt{2}}\big (|n/2,n/2\rangle +\text {e}^{in\varphi }|n/2,-n/2\rangle \big ), \end{aligned}$$

where $\{|j,m\rangle \}$ for $m=-j,...,j$ denotes the collective basis constructed from the eigenvectors of $J_{z}$, representing the *z* component of the total angular momentum for all qubits, i.e., $J_{z}|j,m\rangle =m|j,m\rangle$. The effect of the collisional dephasing channel on input state (65), can be modeled as the interaction between the qubits and the common thermal reservoir^59,60^. In the interaction picture, the master equation of the system can be expressed as^61^

66 $$\begin{aligned} {\dot{\rho }}(t)=\frac{\gamma }{2}[2J_{z}\rho (t)J_{z}-\rho (t)J_{z}^{2} -J_{z}^{2}\rho (t)], \end{aligned}$$

where $\gamma$ represents the dephasing rate which may be time-dependent, and $\rho$ denotes the reduced density operator of the system. In the case of single-qubit systems, Eq. (66) reduces to

67 $$\begin{aligned} {\dot{\rho }}(t)=\frac{\gamma }{2}[\sigma _{z}\rho (t)\sigma _{z}-\rho (t)], \end{aligned}$$

corresponding to a single-qubit dephasing channel. Because the QFI and the HSS remain invariant under the unitary evolution being independent of the phase parameter, working in the interaction picture does not affect the result of the calculation. Solving Eq. (82), one can find that the time evolution of the density matrix elements is expressed as

68 $$\begin{aligned} \rho _{m,m'}(t)\equiv \langle j,m|\rho (t)|j,m'\rangle =\rho _{m,m'}(0)\text {e}^{-(m-m')^{2}\tau }, \end{aligned}$$

where $$\tau =\gamma t$$.

We are interested to compare the dynamics of the QFI and HSS when computed with respect to initial phase ${\widetilde{\varphi }}= n\varphi$ imprinted on the input state (65). The corresponding QFI is given by^60^

69 $$\begin{aligned} F_{{\widetilde{\varphi }}}=n\text {e}^{-2n^{2}\tau }. \end{aligned}$$

In addition, we find that the HSS has the following simple exponential form:

70 $$\begin{aligned} HSS_{{\widetilde{\varphi }}}=\dfrac{{{\text {e}}^{-2\, \left( {2}^{n}-1 \right) ^{2}\tau }}}{2}, \end{aligned}$$

resulting in the relation

71 $$\begin{aligned} F_{{\widetilde{\varphi }}}= n{{\text {e}}^{{\frac{{n}^{2}\ln \left( 2\,{HSS_{{\widetilde{\varphi }}}} \right) }{ \left( { 2}^{n}-1 \right) ^{2}}}}} \Longrightarrow \dfrac{\text {d}F_{{\widetilde{\varphi }}}}{\text {d}t}={\frac{{n}^{3}}{{HSS_{{\widetilde{\varphi }}}}\, \left( {2}^{n}-1 \right) ^{2}}{{\text {e}}^{{ \frac{{n}^{2}\ln \left( 2\,{HSS_{{\widetilde{\varphi }}}} \right) }{ \left( {2}^{n}-1 \right) ^{2}}}}}} \dfrac{\text {d}HSS_{{\widetilde{\varphi }}}}{\text {d}t}, \end{aligned}$$

by which our main result can be verified straightforwardly.

## Discussion

Quantum information processing based on *n*-qubit registers provides a paradigmatic playground for fundamental research on composite quantum systems with potential technological advances^50,62–65^. For this reason, finding useful tools for the convenient characterization of quantum features within quantum registers is particularly desired.

In this paper, we have constructed a strong relationship for *n*-qubit systems between the Hilbert–Schmidt speed (HSS), which is a special case of quantum statistical speed, and the quantum Fisher information (QFI), a key concept in parameter estimation theory and in quantum metrology in general. The idea underlying this relationship stems from the fact that the QFI, quantifying the sensitivity of an initial state with respect to changes of the parameter of a dynamical evolution, is itself a quantum statistical speed extracted from the Bures distance. In fact, our detailed analysis, carried out for several paradigmatic open quantum systems ranging from one qubit to *n* qubits under different environmental conditions, shows that these two quantities exhibit the same qualitative dynamical behaviors. Therefore, in contrast to the typical computational complication of the QFI, especially for multipartite systems, our findings provide evidence that the HSS can be instead adopted as an efficient tool in the process of quantum phase estimation implemented by *n*-qubit systems, because of its straightforward determination.

We point out that the present results are an advance of previously reported analysis^66^, where the HSS has been shown to be useful for metrological resolution of phase shift induced by a unitary transformation without noisy dynamics. Here, instead, we provide evidence that the HSS is a powerful figure of merit for the enhanced estimation of a phase encoded in the initial state of an open system made of *n* qubits subject to noisy channels. As an interesting outlook, these results suggest to extend the analysis to problems in noisy quantum phase estimation, which encompass cases when both parameter encoding and noisy dynamics happen simultaneously (as occurs, for instance, in frequency estimation processes under pure dephasing^67^).

On the one hand, it is known that the QFI monotonically decreases under Markovian dynamics, since it cannot increase under completely positive maps^68–70^, so that it can be used as a witness of non-Markovianity. Originally, introducing a flow of QFI as ${\mathcal {I}}_{\varphi }(t)=\text {d}F_{\varphi }(t)/{\text {d}t}$, it has been suggested^71^ that if ${\mathcal {I}}_{\varphi }(t)>0$ for some *t*, then the time evolution is non-Markovian. Nevertheless, the efficiency of the QFI flow to detect the non-Markovianity in various scenarios has not been yet analytically compared with the other faithful witnesses of non-Markovianity (see Ref.^72^ for a numerical study on the interplay between the dynamics of the QFI and the *trace distance* an important indicator of non-Markovianity). On the other hand, the HSS flow ${\text {d}HSS_{\varphi }(t)}/{\text {d}t}$ has been recently proposed as a faithful witness of non-Markovianity in low dimensional systems^73,74^. As a useful consequence, our results also provide a sanity check of the QFI flow as a witness of non-Markovianity. Moreover, because the QFI is always contractive under Markovian dynamics, our results supply strong evidence for contractivity of the HSS under memoryless evolution of high-dimensional systems and pave the way to further studies on its applications in measuring the non-Markovianity in open quantum systems made of qudits.

Finally, it should be noted that much attention has been recently devoted to exploring the use of continuous variable (CV) systems in quantum information processing^75–77^. It originates from the fact that the continuous-spectrum quantum variables can be easier to manipulate than quantum bits for performing various quantum information processes. Therefore, generalizing our results to the applicability of HSS in the CV context remains of interest and will be addressed elsewhere.

## References

[1] Giovannetti V, Lloyd S, Maccone L (2006). Quantum metrology. Phys. Rev. Lett..

[2] Giovannetti V, Lloyd S, Maccone L (2004). Quantum-enhanced measurements: Beating the standard quantum limit. Science.

[3] Holevo A (1978). Estimation of shift parameters of a quantum state. Rep. Math. Phys..

[4] Paris MG (2009). Quantum estimation for quantum technology. Int. J. Quantum Inf..

[5] Giovannetti V, Lloyd S, Maccone L (2011). Advances in quantum metrology. Nat. Photon..

[6] Liu J, Yuan H, Lu X-M, Wang X (2019). Quantum fisher information matrix and multiparameter estimation. J. Phys. A.

[7] Tóth G, Apellaniz I (2014). Quantum metrology from a quantum information science perspective. J. Phys. A.

[8] Jafarzadeh M, Rangani Jahromi H, Amniat-Talab M (2020). Effects of partial measurements on quantum resources and quantum Fisher information of a teleported state in a relativistic scenario. Proc. R. Soc. A.

[9] Rangani Jahromi H (2020). Quantum thermometry in a squeezed thermal bath. Phys. Scr..

[10] Polino E, Valeri M, Spagnolo N, Sciarrino F (2020). Photonic quantum metrology. AVS Quantum. Science.

[11] Pirandola S, Bardhan BR, Gehring T, Weedbrook C, Lloyd S (2018). Advances in photonic quantum sensing. Nat. Photon..

[12] Haase JF, Smirne A, Huelga SF, Kolodynski J, Demkowicz-Dobrzanski R (2018). Precision limits in quantum metrology with open quantum systems. Quant. Meas. Quant. Metrol..

[13] Bongs K (2019). Taking atom interferometric quantum sensors from the laboratory to real-world applications. Nat. Rev. Phys..

[14] Pezzè L, Smerzi A, Oberthaler MK, Schmied R, Treutlein P (2018). Quantum metrology with nonclassical states of atomic ensembles. Rev. Mod. Phys..

[15] Castellini A (2019). Indistinguishability-enabled coherence for quantum metrology. Phys. Rev. A.

[16] Abbott BP (2016). Observation of gravitational waves from a binary black hole merger. Phys. Rev. Lett..

[17] Tse M (2019). Quantum-enhanced advanced ligo detectors in the era of gravitational-wave astronomy. Phys. Rev. Lett..

[18] Acernese F (2019). Increasing the astrophysical reach of the advanced virgo detector via the application of squeezed vacuum states of light. Phys. Rev. Lett..

[19] Haocun Y (2020). Quantum correlations between light and the kilogram-mass mirrors of ligo. Nature.

[20] Mason D, Chen J, Rossi M, Tsaturyan Y, Schliesser A (2019). Continuous force and displacement measurement below the standard quantum limit. Nat. Phys..

[21] Huelga SF (1997). Improvement of frequency standards with quantum entanglement. Phys. Rev. Lett..

[22] Kasevich M, Chu S (1992). Measurement of the gravitational acceleration of an atom with a light-pulse atom interferometer. Appl. Phys. B.

[23] Ménoret V (2018). Gravity measurements below $$10^{- 9} g$$ with a transportable absolute quantum gravimeter. Sci. Rep..

[24] Taylor MA (2013). Biological measurement beyond the quantum limit. Nat. Photonics.

[25] Pitkin M, Reid S, Rowan S, Hough J (2011). Gravitational wave detection by interferometry (ground and space). Living Rev. Relativ..

[26] Dorner U (2009). Optimal quantum phase estimation. Phys. Rev. Lett..

[27] Helstrom CW (1976). Quantum detection and estimation theory.

[28] Braunstein SL, Caves CM (1994). Statistical distance and the geometry of quantum states. Phys. Rev. Lett..

[29] Braunstein SL, Caves CM, Milburn G (1996). Generalized uncertainty relations: Theory, examples, and Lorentz invariance. Ann. Phys..

[30] Gessner M, Smerzi A (2018). Statistical speed of quantum states: Generalized quantum fisher information and schatten speed. Phys. Rev. A.

[31] Jeffreys H (1946). An invariant form for the prior probability in estimation problems. Proc. R. Soc. Lond. A.

[32] Šafránek D (2018). Simple expression for the quantum Fisher information matrix. Phys. Rev. A.

[33] Luo S, Zhang Q (2004). Informational distance on quantum-state space. Phys. Rev. A.

[34] Jozsa R (1994). Fidelity for mixed quantum states. J. Mod. Opt..

[35] Ozawa M (2000). Entanglement measures and the Hilbert–Schmidt distance. Phys. Lett. A.

[36] Breuer H, Petruccione F, Petruccione S (2002). The Theory of Open Quantum Systems.

[37] Haikka P, Johnson T, Maniscalco S (2013). Non-Markovianity of local dephasing channels and time-invariant discord. Phys. Rev. A.

[38] Bellomo B, Lo Franco R, Compagno G (2007). Non-markovian effects on the dynamics of entanglement. Phys. Rev. Lett..

[39] Bellomo B, Lo Franco R, Compagno G (2008). Entanglement dynamics of two independent qubits in environments with and without memory. Phys. Rev. A.

[40] Lo Franco R, Bellomo B, Maniscalco S, Compagno G (2013). Dynamics of quantum correlations in two-qubit systems within non-Markovian environments. Int. J. Mod. Phys. B.

[41] Maniscalco S, Francica F, Zaffino RL, Lo Gullo N, Plastina F (2008). Protecting entanglement via the quantum Zeno effect. Phys. Rev. Lett..

[42] Lee J, Kim MS (2000). Entanglement teleportation via Werner states. Phys. Rev. Lett..

[43] Bowen G, Bose S (2001). Teleportation as a depolarizing quantum channel, relative entropy, and classical capacity. Phys. Rev. Lett..

[44] Rangani Jahromi H, Amini M, Ghanaatian M (2019). Multiparameter estimation, lower bound on quantum fisher information, and non-markovianity witnesses of noisy two-qubit systems. Quantum Inf. Process..

[45] Lombardo FC, Villar PI (2010). Environmentally induced effects on a bipartite two-level system: Geometric phase and entanglement properties. Phys. Rev. A.

[46] Ho S-H, Chao S-P, Chou C-H, Lin F-L (2014). Decoherence patterns of topological qubits from Majorana modes. New J. Phys..

[47] Rangani Jahromi H, Haseli S (2020). Quantum memory and quantum correlations of Majorana qubits used for magnetometry. Quantum Inf. Comput..

[48] Leung DW (2003). Choi’s proof as a recipe for quantum process tomography. J. Math. Phys..

[49] Nielsen MA, Chuang IL (2010). Quantum computation and quantum information.

[50] Horodecki R, Horodecki P, Horodecki M, Horodecki K (2009). Quantum entanglement. Rev. Mod. Phys..

[51] Greenberger DM, Horne MA, Shimony A, Zeilinger A (1990). Bell’s theorem without inequalities. Am. J. Phys..

[52] Bužek V, Hillery M (1998). Universal optimal cloning of arbitrary quantum states: from qubits to quantum registers. Phys. Rev. Lett..

[53] Braunstein SL (1999). Separability of very noisy mixed states and implications for nmr quantum computing. Phys. Rev. Lett..

[54] Shang J, Gühne O (2018). Convex optimization over classes of multiparticle entanglement. Phys. Rev. Lett..

[55] Cohn I, De Oliveira ALF, Buksman E, De Lacalle JGL (2016). Grover’s search with local and total depolarizing channel errors: Complexity analysis. Int. J. Quant. Inform..

[56] Briegel H-J, Dür W, Cirac JI, Zoller P (1998). Quantum repeaters: the role of imperfect local operations in quantum communication. Phys. Rev. Lett..

[57] Xiao X, Yao Y, Zhou L-M, Wang X (2014). Distribution of quantum Fisher information in asymmetric cloning machines. Sci. Rep.

[58] Romero KF, Franco RL (2012). Simple non-Markovian microscopic models for the depolarizing channel of a single qubit. Phys. Scr.

[59] Bar-Gill N, Rao DB, Kurizki G (2011). Creating nonclassical states of Bose–Einstein condensates by dephasing collisions. Phys. Rev. Lett..

[60] Zhong W, Sun Z, Ma J, Wang X, Nori F (2013). Fisher information under decoherence in Bloch representation. Phys. Rev. A.

[61] Dorner U (2012). Quantum frequency estimation with trapped ions and atoms. New J. Phys..

[62] Bradley CE (2019). A ten-qubit solid-state spin register with quantum memory up to one minute. Phys. Rev. X.

[63] Schrader D (2004). Neutral atom quantum register. Phys. Rev. Lett..

[64] Robledo L (2011). High-fidelity projective read-out of a solid-state spin quantum register. Nature.

[65] King BE (1998). Cooling the collective motion of trapped ions to initialize a quantum register. Phys. Rev. Lett..

[66] Rivas Á, Luis A (2008). Intrinsic metrological resolution as a distance measure and nonclassical light. Phy. Rev. A.

[67] Huelga SF (1997). Improvement of frequency standards with quantum entanglement. Phys. Rev. Lett..

[68] Fujiwara A (2001). Quantum channel identification problem. Phys. Rev. A.

[69] Suzuki J (2016). Entanglement detection and parameter estimation of quantum channels. Phys. Rev. A.

[70] Laurenza R, Lupo C, Spedalieri G, Braunstein SL, Pirandola S (2018). Channel simulation in quantum metrology. Quantum Meas. Quantum Metrol..

[71] Lu X-M, Wang X, Sun C (2010). Quantum fisher information flow and non-Markovian processes of open systems. Phys. Rev. A.

[72] Mirkin N, Larocca M, Wisniacki D (2020). Quantum metrology in a non-Markovian quantum evolution. Phy. Rev. A.

[73] Rangani Jahromi H, Mahdavipour K, Khazaei Shadfar M, Lo Franco R (2020). Witnessing non-Markovian effects of quantum processes through Hilbert-Schmidt speed. Phys. Rev. A.

[74] Rangani Jahromi, H., Nori, F. & Lo Franco, R. Witnessing non-Markovianity and criticality in (anti-) parity-time-symmetric systems. arXiv:2101.04663 (2021).

[75] Braunstein SL, Van Loock P (2005). Quantum information with continuous variables. Rev. Mod. Phys..

[76] Braunstein S. L, Pati A. K (2012). Quantum information with continuous variables.

[77] Ferraro, A., Olivares, S. & Paris, M. G. Gaussian states in continuous variable quantum information. arXiv:0503237 [quant-ph] (2005).

